# Genomic Characterization of Extended-Spectrum β-Lactamase-Producing and Third-Generation Cephalosporin-Resistant *Escherichia coli* Isolated from Stools of Primary Healthcare Patients in Ethiopia

**DOI:** 10.3390/antibiotics13090851

**Published:** 2024-09-05

**Authors:** Deneke Wolde, Tadesse Eguale, Girmay Medhin, Aklilu Feleke Haile, Haile Alemayehu, Adane Mihret, Mateja Pirs, Katja Strašek Smrdel, Jana Avberšek, Darja Kušar, Tjaša Cerar Kišek, Tea Janko, Andrej Steyer, Marjanca Starčič Erjavec

**Affiliations:** 1 Department of Medical Laboratory Science, College of Medicine and Health Sciences, Wachemo University, Hossana P.O. Box 667, Ethiopia; deneke2024@wcu.edu.et; 2 Aklilu Lemma Institute of Pathobiology, Addis Ababa University, Addis Ababa P.O. Box 1176, Ethiopia; tadesse.eguale@aau.edu.et (T.E.); girmay.medhin@aau.edu.et (G.M.); aklilu.feleke@aau.edu.et (A.F.H.); haile.alemayehu@aau.edu.et (H.A.); 3 Department of Microbiology, Biotechnical Faculty, University of Ljubljana, 1000 Ljubljana, Slovenia; 4 Ohio State Global One Health, Addis Ababa, Ethiopia; 5 College of Health Sciences, Addis Ababa University, Addis Ababa P.O. Box 1176, Ethiopia; adane.mihret@aau.edu.et; 6 Armauer Hansen Research Institute, Addis Ababa P.O. Box 1005, Ethiopia; 7 Institute of Microbiology and Immunology, Faculty of Medicine, University of Ljubljana, 1000 Ljubljana, Slovenia; mateja.pirs@mf.uni-lj.si (M.P.); katja.strasek@mf.uni-lj.si (K.S.S.); 8 Institute of Microbiology and Parasitology, Veterinary Faculty, University of Ljubljana, 1000 Ljubljana, Slovenia; jana.avbersek@vf.uni-lj.si (J.A.); darja.kusar@vf.uni-lj.si (D.K.); 9 National Laboratory of Health, Environment and Food, 2000 Maribor, Slovenia; tjasa.cerar.kisek@nlzoh.si (T.C.K.); tea.janko@nlzoh.si (T.J.); andrej.steyer@nlzoh.si (A.S.)

**Keywords:** *Escherichia coli*, antimicrobial resistance genes, virulence-associated genes, mobile genetic elements, phylogenetic groups, Ethiopia

## Abstract

The global spread of antimicrobial resistance genes (ARGs) in *Escherichia coli* is a major public health concern. The aim of this study was to investigate the genomic characteristics of extended-spectrum β-lactamase (ESBL)-producing and third-generation cephalosporin-resistant *E. coli* from a previously obtained collection of 260 *E. coli* isolates from fecal samples of patients attending primary healthcare facilities in Addis Ababa and Hossana, Ethiopia. A total of 29 *E. coli* isolates (19 phenotypically confirmed ESBL-producing and 10 third-generation cephalosporin-resistant isolates) were used. Whole-genome sequencing (NextSeq 2000 system, Illumina) and bioinformatic analysis (using online available tools) were performed to identify ARGs, virulence-associated genes (VAGs), mobile genetic elements (MGEs), serotypes, sequence types (STs), phylogeny and conjugative elements harbored by these isolates. A total of 7 phylogenetic groups, 22 STs, including ST131, and 23 serotypes with different VAGs were identified. A total of 31 different acquired ARGs and 10 chromosomal mutations in quinolone resistance-determining regions (QRDRs) were detected. The isolates harbored diverse types of MGEs, with *IncF* plasmids being the most prevalent (66.7%). Genetic determinants associated with conjugative transfer were identified in 75.9% of the *E. coli* isolates studied. In conclusion, the isolates exhibited considerable genetic diversity and showed a high potential for transferability of ARGs and VAGs. Bioinformatic analyses also revealed that the isolates exhibited substantial genetic diversity in phylogenetic groups, sequence types (ST) and serogroups and were harboring a variety of virulence-associated genes (VAGs). Thus, the studied isolates have a high potential for transferability of ARGs and VAGs.

## 1. Introduction

Antimicrobial resistance (AMR) is a major global health problem that poses a significant threat to public health [1]. It is rapidly increasing and is expected to result in high healthcare costs and poor patient outcomes, potentially making AMR the leading cause of global mortality [2]. Africa and Asia are the two regions highly affected by AMR, with the potential for over 4.1 million annual deaths from AMR by 2050 [3]. *Escherichia coli* is one of the most important human pathogens and represents a major public health challenge. It is also one of the widely abundant commensal organisms in the gastrointestinal tracts of humans and animals, known for its ability to easily develop resistance to antimicrobial agents and to serve as a vector for the spread of AMR, which is a major problem for humans and animals [4]. In Ethiopia, there is an emergence and spread of resistance to common antimicrobials among *E. coli* isolates, leading to increased morbidity, mortality, and healthcare costs [5].

Infections with *E. coli* resistant to critically important antimicrobials, such as third-generation cephalosporins, have increased worldwide [6,7] and pose a challenge to clinical antibiotic therapy. *E. coli* strains resistant to third-generation cephalosporin antibiotics are frequently associated with the expression of extended-spectrum β-lactamases (ESBLs) [8]. ESBL production is the most problematic mechanism of antibiotic resistance, as many β-lactamase-encoding genes are typically carried on mobile genetic elements, such as plasmids, which can be easily transferred between different bacterial species [9]. Resistance to third-generation cephalosporins in *E. coli* can complicate the treatment of infections and lead to the use of a last-resort antimicrobial class such as carbapenems [10], which are not readily available in developing countries.

Bacterial populations acquire resistance to antibiotics either through chromosomal mutation or through horizontal gene transfer (HGT) from other bacteria that are either distant or closely related. This transfer is facilitated by mobile genetic elements (MGEs) [11]. MGEs are DNA elements that can move within or between bacterial cells, either from a chromosome to a plasmid or between plasmids through non-conjugative transposons, gene cassettes, and insertion sequence elements, or between DNA molecules through MGE elements capable of self-replication and conjugative transfer, such as plasmids and integrative conjugative elements [12].

MGEs carry genes that can confer resistance to antibiotics and play a critical role in facilitating the acquisition and spread of antimicrobial resistance genes (ARGs) through HGT [13]. The main mechanisms of HGT are conjugation, transduction, and transformation [14]. Conjugation drives the rapid evolution and adaptation of bacterial strains by facilitating the spread of diverse metabolic capabilities, including virulence, biofilm formation, and heavy metal and antibiotic resistance [15]. The conjugative transfer regions of the self-transmissible MGEs typically involve an origin of transfer (*oriT*) region, a conjugative type IV secretion system (T4SS), type IV coupling protein (ATPase), and a relaxase to deliver single-stranded DNA (ssDNA) into the recipient cell [16]. Understanding the profile of virulence-associated genes (VAGs), ARGs, and MGEs among *E. coli* strains circulating in a certain region is important for preventing the dissemination of antibiotic resistance, virulence factors, and other clinically relevant traits through *E. coli* bacterial populations. As there have been only a few studies conducted on this topic in Ethiopia, the aim of this study was to assess the distribution and diversity of ARGs, VAGs, MGEs, sequence types, serotypes and conjugative elements in 29 phenotypically confirmed ESBL-producing and/or third-generation cephalosporin-resistant *E. coli* isolates, obtained from patients attending primary healthcare units in Addis Ababa (*n* = 11) and Hossana (*n* = 18) using whole-genome sequencing.

## 2. Results

### 2.1. Phylogenetic Groups of *E. coli* Isolates

**Figure 1:**
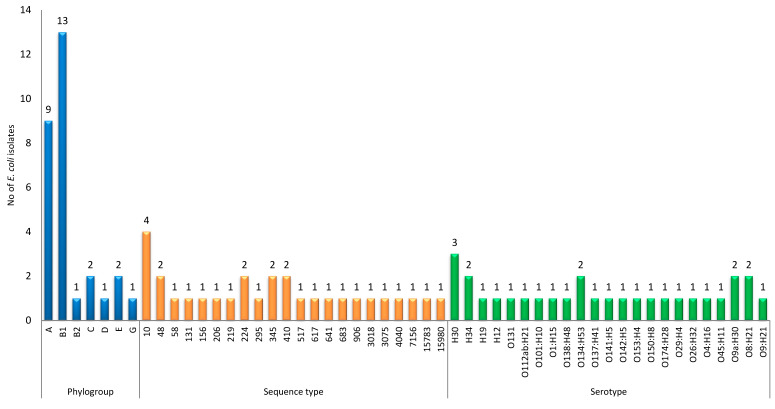
Phylogroups, sequence types, and serotypes of studied *E. coli* isolates.

The *E. coli* isolates analyzed in this study belonged to seven phylogenetic groups. The predominant phylogenetic group was phylogroup B1 (*n* = 13; 44.8%), followed by phylogroup A (*n* = 9; 31.03%). The other phylogenetic groups identified in this study were phylogroup C and E (*n* = 2 each), phylogroup B2 (*n* = 1), phylogroup D (*n* = 1), and phylogroup G (*n* = 1) (Figure 1). Phylogroups B2, C, D, and G were obtained from both non-diarrheic and diarrheic patients from Hossana. These phylogroups were found in a slightly higher proportion (60%) among females, and except for one of the phylogroup C isolate, all of them were detected in the age groups of 5–9 and 20–45 years (Table 1).

### 2.2. Sequence Types of *E. coli* Isolates

Twenty-two different STs were identified, of which one was a novel sequence type designated as ST15980. The most frequently identified ST was ST10 (*n* = 4; 13.8%), followed by ST48, ST224, ST345, and ST410 (*n* = 2; 6.9% each). ST131 was also detected in this study in one *E. coli* isolate obtained from a non-diarrheic patient in Hossana. The novel ST was also among isolates from Hossana. Of the total STs identified, only ST10 and ST48 were detected in *E. coli* isolates from both Addis Ababa and Hossana (Figure 1 and Supplementary Table S1).

### 2.3. Serotypes of *E. coli* Isolates 

A total of 23 different serotypes were recorded among the 29 *E. coli* isolates investigated in this study. Of the total number of tested isolates, 22 (75.8%) were typable for their O antigen and 28 (96.5%) for their H antigen, with 7 (24.1%) being ‘rough’ (lacking expression of the O antigen) and 1 (3.4%) lacking expression of the H antigen. The most frequently identified serotype was H30 (*n* = 3; 10.3%), followed by four serotypes, namely H34, O134:H53, O8:H21, and O9a:H30, each represented by two isolates (Figure 1). The serotype O153:H4 detected in this study was identified as ST131 (Supplementary Table S1).

### 2.4. Virulence-Associated Gene Profile of *E. coli* Isolates

**Figure 2:**
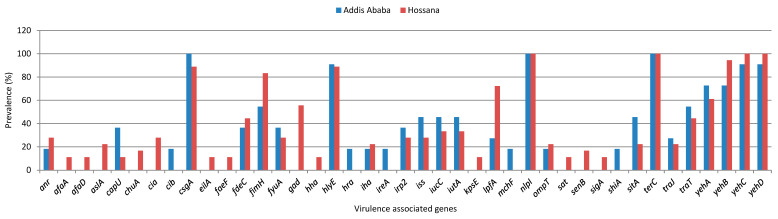
Virulence-associated genes detected with prevalence greater than 10% in studied *E. coli* isolates from Addis Ababa and Hossana.

A total of 72 different VAGs involved in adhesion (*n* = 23), protection against environmental stress and host immune response (*n* = 32), toxin production (*n* = 9), and in iron acquisition (*n* = 8) were identified in the *E. coli* isolates tested in this study. On average, all *E. coli* isolates carried 16 different VAGs, with a minimum of 7 and a maximum of 28 VAGs. The *terC* gene, which encodes for the tellurite resistance, and the *nlpI* gene, which encodes an outer membrane lipoprotein, were identified in all isolates tested. A total of 28 (96.6%) of the isolates contained *yehC* (chaperone, YhcD fimbrial cluster), 27 (93.1%) *csgA* (curlin major subunit CsgA), 26 (89.7%) *hlyE* (avian *E. coli* haemolysin), and 25 (86.2%) *yehB* (usher, YhcD fimbrial cluster). Forty-one of the total virulence-associated genes were detected in isolates from Hossana and Addis Ababa (Supplementary Table S1). Only seven genes, including the *cdt-IIIB* gene, which encodes the CdtB subunit of the cytolethal distending toxin complex, *cib* gene, which encodes a protein that protects *E. coli* cells from the cytotoxic effects of the cloacin DF13 bacteriocin, *colE5* (colicin E5 lysis protein Lys), *etsC* (putative type I secretory outer membrane protein) and *mcbA* (bacteriocin microcin B17) were detected exclusively in isolates from Addis Ababa. However, 27 different genes, including capsular polysaccharide-related genes (*kpsE*, *kpsMII*, and *kpsMII_K5*), the *tia* gene that encodes the Tia (toxigenic invasion locus) protein, which is involved in the adherence and invasion of host epithelial cells, and the *usp* (uropathogenic specific protein), were exclusively detected in isolates from Hossana (Supplementary Table S1). The most frequently detected VAGs (greater than 10%) in *E. coli* isolates from Addis Ababa and Hossana are presented in Figure 2. The study found that 28 VAGs were detected in 18.2% to 100% of the *E. coli* isolates from Addis Ababa, while 36 VAGs were detected in 11.1% to 100% of the isolates from Hossana.

The *afaB*, *afaC*, *afaE*, and *kpsMII*_*K5* genes were only found in the O153:H4 serotype. This serotype also possessed genes involved in the biosynthesis and uptake of yersiniabactin, aerobactin, and salmochelin siderophores. Seven VAGs (*fimH*, *nlpI*, *terC*, *yehA*, *yehB*, *yehC* and *yehD*) were detected in isolates from all phylogenetic groups. The *afaB*, *afaC*, *afaE*, and *kpsMII_K5* genes were only detected in phylogroup B2 isolates. Fourteen genes, including *usp*, *aslA*, *sigA*, *traT*, *hlyE*, *gad*, *chuA*, *cia*, *csgA*, *iha*, *ireA*, *iucC*, *iutA*, and *ompT*, were detected in phylogroup G. The *neuC* gene, which encodes a polysialic acid capsule biosynthesis protein, the *kpsMII* gene, which encodes a polysialic acid transport protein group 2 capsule and the *usp* gene, which encodes a uropathogenic specific protein, were only detected in phylogroups C, D, and G, respectively. Twenty-eight different VAGs were detected in a single ST131 *E. coli* isolate, among them the *sat* gene, which encodes the serine protease autotransporters of *Enterobacteriaceae* (SPATE), and the *senB* gene, which encodes the plasmid-encoded enterotoxin (Supplementary Table S1).

### 2.5. Antimicrobial Resistance Genes

The distribution of ARGs among *E. coli* isolates is presented in Supplementary Table S2. ARGs were detected in 28 (96.6%) of *E. coli* isolates tested in this study. Thirty-one different horizontally transmitted acquired ARGs and ten chromosomal mutations in quinolone resistance-determining regions (QRDRs) were detected in *E. coli* isolates. Of the total *E. coli* isolates that carried ARGs, 19 (67.9%) had 5 or more different ARGs. The most prevalent ARGs detected were *bla*_CTX-M-15_ (*n* = 22 isolates), followed by *bla*_TEM-1B_ (*n* = 15 isolates), and *tet*(A) (*n* = 14 isolates). The *bla*_CTX-M-15_ gene was identified in 78.9% of phenotypically confirmed ESBL-producing and 70% of third-generation cephalosporin-resistant *E. coli*. In total, 2 *E. coli* isolates from Hossana carried 14 different resistance genes encoding for resistance to antibiotics of different classes. Among third-generation cephalosporin-resistant *E. coli*, 90% carried β-lactamase and quinolone resistance genes. In this study, the plasmid-mediated quinolone resistance (PMQR) determinants, such as *qnrS1*, *aac(6*′*)-Ib-cr*, *qepA1*, *qepA2*, and *qepA4*, were detected. Among these, *qnrS1* was the most predominant, being detected in 13 (44.8%) of the isolates. Mutations in the QRDR and PMQR genes co-existed in 5 (20%) of the *E. coli* isolates carrying genes that determine resistance to quinolones. Of the *E. coli* isolates tested, 48.3% had chromosomal mutations in *gyrA*, *parC* and/or *parE*, which are associated with resistance to quinolones—ciprofloxacin and nalidixic acid. Double substitutions in *gyrA* (S83L + D87N) were observed in 8 (27.6%) of the *E. coli* isolates and these isolates also had an additional substitution in the *parC* (S80I) and *parE* (S458A) genes. Single amino acid substitutions, including S83L (*n* = 3), S83A (*n* = 1) and S83V (*n* = 1), were also observed in the *gyrA* gene. The substitutions at S80I (*n* = 6), E84K (*n* = 1), and A56T (*n* = 1) in *par*C, as well as S458A (*n* = 7), L416F (*n* = 1), and I529L (*n* = 1) in *parE*, were also observed (Table 2 and Supplementary Table S2).

This study found 7 *E. coli* serotypes (H30, O138:H48, O101:H10, O174:H28, O134:H53, O9a:H30, O8:H21) harboring 10 to 14 different ARGs. The predominant serotypes carrying the highest number (14) of ARGs were O9a:H30 and O8:H21. Furthermore, the *E. coli* isolates across all phylogenetic groups, with the exception of 1 from group B1, carried a range of ARGs, with groups A and B1 sharing 24 different ARGs. The analysis of sequence types revealed that ST224 and ST410 *E. coli* carried a high proportion of ARGs (Supplementary Table S2).

### 2.6. Co-Occurrence of Antimicrobial Resistance Genes 

Several ARGs were detected together in most of the isolates in the current study. All *E. coli* isolates carrying at least one aminoglycoside antibiotic resistance gene were shown to carry β-lactam, quinolone, sulfonamide, and trimethoprim resistance genes simultaneously. *E. coli* isolates that carried at least one β-lactam antibiotic resistance gene also carried quinolone resistance genes and sulfonamide resistance genes in 89.3% and 75% of the cases, respectively. The β-lactam antibiotic resistance genes were also detected in 39.3% of *E. coli* isolates that carried at least one trimethoprim resistance gene, 32.1% of *E. coli* isolates that carried at least one macrolide resistance gene and 10.7% of *E. coli* isolates that carried at least one amphenicol resistance gene (Supplementary Table S2).

### 2.7. Plasmids and Other Mobile Genetic Elements

**Figure 3:**
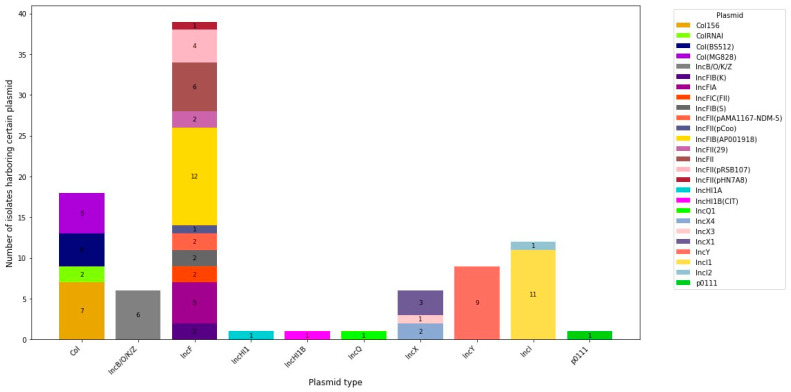
Number of studied *E. coli* isolates harboring certain plasmid types.

In this study, *E. coli* isolates were found to harbor diverse MGEs, including plasmids, insertion sequences (IS), integrative conjugative elements (ICEs), and transposons. A total of 86 MGEs were detected in the *E. coli* isolates. Among these, 26 were identified as plasmids, 50 as IS, and 6 as composite transposons and 2 as unit transposons. The remaining two were ICEs and miniature inverted repeats. Except for one isolate, all tested *E. coli* isolates were confirmed to carry one to eleven plasmids. The *IncF* plasmid was the most abundant plasmid in *E. coli* isolates, with different replicon types and replicon variants, including *IncFII*, *IncFII(29)*, *IncFIB(AP001918)*, *IncFII(pRSB107)*, *IncFII(pAMA1167-NDM-5)*, *IncFII(pHN7A8)*, *IncFII(pCoo)*, *IncFIB(S)*, *IncFIC(FII)*, *IncFIA(HI1)*, *IncFII(pSE11)*, *IncFIA*, and *IncFIB(K)*. The isolates also carried other incompatibility group plasmids, including *IncI1* (*n* = 11), *IncI2* (*n* = 1), *IncY* (*n* = 9), *IncX1* (*n* = 3), *IncX3* (*n* = 1), and *IncX4* (*n* = 2). The *Col* plasmids detected in the *E. coli* isolates in this study were *Col156* (*n* = 7), *Col(MG828)* (*n* = 5), *ColRNAI* (*n* = 2), and *Col(BS512)* (*n* = 4). A total of 26 plasmids’ combination patterns were detected in *E. coli* isolates. Nine isolates carried two plasmids, while four isolates carried three plasmids. Nine isolates carried four or more plasmids, while a single isolate obtained from patient at Hossana carried eleven plasmids (Figure 3).

A total of 24 different plasmids were detected in *E. coli* isolates carrying genes resistant to quinolones, while 22 plasmids were detected in *E. coli* isolates carrying genes resistant to antibiotics of the aminoglycoside class. The *IncI2* plasmid was detected only in *E. coli* isolates harboring genes with resistance to the quinolones, whereas the *IncHI1A* and *IncHI1B(CIT)* plasmids were detected only in *E. coli* isolates harboring genes with resistance to the β-lactams. The dominant plasmid detected in all classes of antibiotic resistance gene-carrying *E. coli* was the *IncFIB(AP001918)* plasmid type.

**Figure 4:**
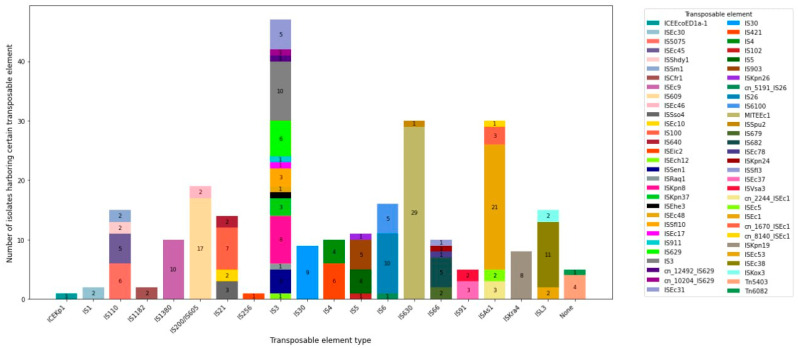
Number of studied *E. coli* isolates harboring certain types of transposable elements.

Similar to plasmids, the *E. coli* isolates also carried several IS elements, miniature inverted repeats, integrative conjugative elements, and transposons. At least four MGEs, other than plasmids, were detected. The MITEEc1 was found in all tested *E. coli* isolates. A total of fifty different IS elements belonging to twenty-one IS families were identified. The most abundant IS family was IS3, followed by ISAs1 and IS630. The most prevalent IS elements were ISEc1 and IS609, detected in 72.4% and 58.6% of all *E. coli* isolates, respectively. Additionally, ISEc38, IS26, and ISEc9 were found in 37.9%, 34.5%, and 34.5% of the isolates, respectively. Isolates carrying twelve different MGEs were the most predominant (14.8%), followed by isolates carrying seven, eight, eleven, and thirteen different MGEs (11.1%) each. Notably, 1 *E. coli* isolate carried 17 different MGEs, while 15 isolates carried 10 to 17 MGEs (Figure 4 and Supplementary Table S3). There was a significant association between genes determining resistance to aminoglycosides and the presence of IS*3* (Fisher exact *p* = 0.01), IS*5075* (Fisher exact *p* = 0.01) and IS*629* (*X2* = 4.821, *p* = 0.028). Similarly, a significant association was observed between genes determining β-lactam resistance and ISEc*31* (Fisher exact *p* = 0.016), ISEc*9* (Fisher exact *p* = 0.048), and ISSfl*8* (Fisher exact *p* = 0.015). In addition, resistance to quinolones was significantly associated with the presence of ISEc*9* (Fisher exact *p* = 0.001) and ISKpn*19* (Fisher exact *p* = 0.003).

### 2.8. Determination of Conjugative Transferable Elements 

Of the 29 *E. coli* isolates tested, 22 (75.9%) had all the essential components necessary for conjugation, such as *oriT*s, relaxases, T4CPs, and T4SSs, indicating their potential for self-transferability. Fourteen (63.6%) of the *E. coli* isolates with conjugative transferable elements were obtained from eight diarrheic patients and six non-diarrheic patients in Hossana, while the remaining eight (three from diarrheic and five from non-diarrheic) were obtained from Addis Ababa. Four (13.8%) of the *E. coli* isolates were found not to carry *oriT*, whereas relaxases were not detected in three (10.3%) isolates. Only T4CP was detected in one *E. coli* isolate; *oriT*, relaxase, and T4SS were absent. Two (6.9%) isolates had both *oriT* and T4CP, but no relaxase and/or T4SS. Twenty-one (95.4%) *E. coli* with conjugative transferable elements had acquired ARGs. Virulence-associated genes were detected in all *E. coli* isolates with self-transferable and non-self-transferable MGEs.

## 3. Discussion

*E. coli* is an extremely diverse bacterial species in which only about 6% of the genes are shared by all strains. The remaining genes, accounting for more than 90%, are variable “accessory genes” that are differentially present in the various *E. coli* strains [17]. The results of the study revealed a high level of genetic diversity among the *E. coli* isolates, which were classified into seven distinct phylogenetic groups. The findings showed that 82.8% of the *E. coli* isolates belonged to phylogroups A, B1, and C, which are generally regarded as phylogenetic groups in which most intestinal pathogenic *E. coli* strains are found in humans [18]. Of all the phylogenetic groups, B1 was the predominant one. This is consistent with previous findings from southwestern Nigeria [19]. In contrast, a study conducted in South Africa among diarrheic children confirmed that a predominant strain characterized by increased virulence and the ability to cause a wide range of infections belonged to phylogroup B2 [20]. The differences can be explained by health, diet and environmental, social, and geographical conditions [21]. The phylogroup B2 *E. coli* strain identified in our study belonged to the ST131. In addition, phylogroup G, which is known to contain highly virulent and AMR strains, was also detected in the current study [22], which implies a higher likelihood of severe and invasive *E. coli* infections.

In the current study, the *E. coli* isolates showed 22 different STs. One of them was a novel ST designated as ST15980, which was isolated from a non-diarrheic patient in Hossana. This isolate showed a broader spectrum of resistance to antimicrobials. The isolate carried resistance genes (*aph(6)-Id*, *aph(3*″*)-Ib*, *bla*_CTX-M-15_, *bla*_TEM-1B_, *qnrS1*, *sul2*, *dfrA14*) conferring resistance to aminoglycosides, β-lactams, quinolones, sulfonamides, and trimethoprim, which complicates treatment with a wide range of common antibiotics and poses a serious health problem. This isolate also typically carried plasmids from incompatibility groups (*IncI1*, *IncY*) and other MGEs such as (*IS26*, *IS30*, *ISEc38*), which often harbor genes conferring resistance to various antibiotics and have the ability to be efficiently transferred between bacterial cells [23]. Many VAGs have also been detected in ST15980, enabling it to adhere to host tissues and cells, evade the host immune system, and cause tissue damage. These include *hlyF*, the gene encoding hemolysin, which may be associated with increased production of outer membrane vesicles and contributes to the release of cytolethal distending toxin and other chemicals [24]; and the *terC* gene, which encodes a subunit of the tellurite resistance protein complex, which may contribute to its fitness and allow it to evade the host’s primary immune response [25], making the strain more virulent and resistant to immune defense.

The prominent ST131, which is a highly virulent and extensively antimicrobial-resistant strain that has spread explosively throughout the world [26], was also found in our study. It is known to cause extraintestinal infections, including urinary tract and bloodstream infections [17]. The ST131 isolated in this study carried 28 VAGs, including *afa* (Dr binding adhesins), *iutA* (aerobactin receptor), and *kpsMT II* (group 2 capsule synthesis), which are characteristic of extraintestinal pathogenic *E. coli* (ExPEC) [18], demonstrating its ability to colonize and persist in the intestine, as confirmed by other studies [19]. Additionally, this isolate contained several genes, including *sat* (secreted autotransporter toxin), *fimH* (type 1 fimbriae), *fyuA* (yersiniabactin receptor), *iha* (adhesin siderophore receptor), *ompT* (outer membrane receptor) *iucC* (aerobactin), *iutA* (aerobactin receptor), and *tratT* (serum resistance associated), that are frequently found in *E. coli* ST131 isolates. Studies have indicated the growing prevalence and spread of the ST131 clonal group in various African countries. In Malawi, a genomic epidemiology study at Queen Elizabeth Central Hospital, a tertiary care center and referral hospital in Lilongwe, found ST131 in 14.9% and 32.8% of the *E. coli* isolates sequenced, respectively [27,28]. Additionally, a study conducted in South Africa found that 18% of *E. coli* isolated from urinary tract infections in inpatients and outpatients belonged to ST131 [29]. Furthermore, a study found that ESBL-producing *E. coli* isolates from Tanzania and Uganda belonged to the ST131 strain, accounting for 8.4% and 2.9% of isolates, respectively [30]. Considering the increased prevalence and pandemic nature of the ST131 strain, which poses a significant threat to public health, due attention should be given to prevent and control its dissemination.

A high degree of diversity was observed in the *E. coli* population with 23 different serotypes. The predominant *E. coli* serotype was H30, which showed increased resistance to different classes of antibiotics. They harbored a plasmid encoding the *bla*_CTX-M-15_ gene, one of the most widespread and predominant ESBL-encoding genes [31]. They also carried genes that determine resistance to aminoglycosides, quinolones, sulfonamides, trimethoprim, tetracyclines, macrolides, and amphenicols. The result also showed that this serotype harbors different groups of plasmids and MGEs containing genes for various VAGs encoding protectins, iron acquisition, cytotoxins, or adhesion factors [32]. The high diversity of ARGs and VAGs and their distribution among various serotypes and STs suggest multiple sources of resistant and virulent bacteria and the flow of these genes between different bacterial populations [33].

Antimicrobial resistance is one of the greatest global threats to public health. The fundamental reason why some bacteria are able to resist the effects of antibiotics is that they have specific genes that code for resistance mechanisms [34]. The resistance genes were detected in 96.6% of the *E. coli* isolates tested in this study. This rate is comparable to a previous report in which resistance genes were found in 84.9% of *E. coli* isolates from humans in the Central Zambia region [35]. However, a lower rate (38%) of ARGs was detected in *E. coli* isolates obtained from human samples in a systematic review and meta-analysis conducted in South Africa [36]. Bioinformatic analysis of the whole-genome sequence of the isolate (MBL protocol number 181) that was found to be phenotypically resistant to ampicillin, cefuroxime, cefotaxime, ceftriaxone, and trimethoprim-sulfamethoxazole did not reveal any ARGs, indicating that this isolate might possess a not yet identified ARG. Another possible explanation could be that the MGE carrying the ARG conferring this specific resistance phenotype was lost in sub-culturing the *E. coli* isolates.

*E. coli* has developed various mechanisms to resist the effects of antibiotics. In many cases, a single strain of *E. coli* can carry ARGs that confer resistance to different classes of antibiotics [37]. This ability significantly complicates the treatment of *E. coli* infections, as clinicians have fewer effective antibiotic options to choose from [38]. The current study found a high co-detection rate of multiple ARGs within the *E. coli* isolates tested. For example, all the isolates carrying aminoglycoside resistance genes were also confirmed to harbor resistance genes for β-lactams, quinolones, sulfonamides, and trimethoprim. Many of the detected resistance genes, including those encoding aminoglycoside-modifying enzymes (AMEs), β-lactamases, and determinants of quinolone resistance, sulfonamides, trimethoprim, and tetracyclines resistance, are often located on MGEs [39,40,41]. Furthermore, plasmids belonging to different incompatibility groups, and other MGEs, which have an important role in the transmission of ARGs, were simultaneously detected in the isolates in the current study. This observation suggests that the diverse MGEs may have the capacity to disseminate different antibiotic classes, further exacerbating the problem of multi-drug-resistant *E. coli* infections and the need to target plasmids to limit acquisition and transmission of antimicrobial resistance, as already reviewed by Vrancianu et al. [42].

Approximately 75.9% of plasmids were found to be self-transferable, possessing key genetic elements such as *oriT*s, relaxases, T4CPs, and T4SSs. This high proportion of self-transferable plasmids indicates their substantial potential for self-transfer and dissemination within bacterial populations. Additionally, it was observed that 95.4% of the conjugative plasmids examined were found to carry acquired ARGs. This suggests that these conjugative plasmids have the capacity to potentially spread and disseminate ARGs among different bacterial species, which poses a significant concern in the context of antimicrobial resistance.

The main limitation of this study was the inability to conclusively determine whether ARGs were plasmid-encoded or chromosomally located. This technical limitation restricts the understanding of the genetic mechanisms underlying the observed multi-drug resistance. Further investigations employing advanced sequencing techniques would be needed to address these limitations and provide a more comprehensive understanding of the antimicrobial resistance landscape in the region.

## 4. Materials and Methods

### 4.1. *E. coli* Isolates 

**Figure 5:**
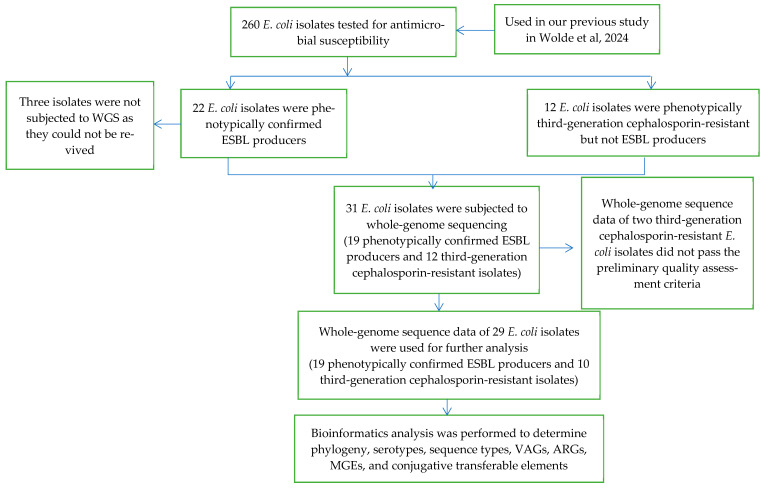
Flow diagram showing the selection of *E. coli* isolates for the study.

The current study is a continuation of a previous cross-sectional study conducted in Addis Ababa and Hossana from October 2021 to September 2022, in which 260 *E. coli* strains were isolated from patients of all ages and sexes recruited according to patient flow from 13 randomly selected health centers from 4 sub-cities (40% of the total) in Addis Ababa and all 4 health centers in Hossana [43]. For the current study, all *E. coli* isolates that showed phenotypic resistance to third-generation cephalosporin antibiotics or were identified as ESBL-producing were selected from the previous collection, because *E. coli* isolates exhibiting these characteristics are critically important for human medicine. The sampling method used in the current study is shown in Figure 5.

### 4.2. DNA Extraction, Whole-Genome Sequencing, Raw Data Pre-Processing, De Novo Assembly, and Quality Control

Twelve selected isolates exhibiting phenotypic resistance to third-generation cephalosporins were subjected to whole-genome sequencing (WGS). Genomic DNA extraction, WGS, raw data pre-processing, de novo assembly and quality control was performed as previously described for the 19 ESBL-producing isolates possessing ESBL genes [43]. The whole-genome sequence data of the two sequenced third-generation cephalosporin-resistant *E. coli* isolates did not pass the preliminary quality assessment criteria and were excluded in further bioinformatic analysis.

### 4.3. Bioinformatics Analysis

The ClermonTyping tool available at http://clermontyping.iame-research.center/ (accessed on 7 June 2024) was used to determine the phylogenetic groups of *E. coli* isolates [44]. Sequence typing was performed based on the seven gene Achtman scheme using the website https://pubmlst.org/ (accessed on 7 June 2024) [45] and EnteroBase https://enterobase.warwick.ac.uk/ (accessed on 27 June 2024) [46]. The detection of *E. coli* virulence-associated genes and serotypes was performed using VirulenceFinder 2.0 and SerotypeFinder available at www.genomicepidemiology.org (accessed on 5 June 2024) with the default parameters [47,48]. In addition, the acquired antimicrobial resistance genes as well as chromosomal point mutations causing resistance were determined using ResFinder 4.0, available at http://genepi.food.dtu.dk/resfinder (accessed on 5 June 2024), with default parameters [49]. Plasmid replicon was identified to infer plasmid presence using PlasmidFinder 2.1, available at https://cge.food.dtu.dk/services/PlasmidFinder/ (accessed on 15 June 2024), with minimum identity of 95% and minimum coverage of 60% [50]. Integrated mobile genetic elements were predicted in the assembled genomes using MobileElementFinder version 1.0 with MGEdb v1.0.2, available at https://cge.food.dtu.dk/services/MobileElementFinder/ (accessed on 15 June 2024) [51].

### 4.4. Statistical Analysis 

The data were captured in Microsoft Excel and analyzed using SPSS software (Version 25. IBM SPSS Inc., New York, NY, USA). Descriptive analyses using frequency and percentages were used to summarize the variables. The chi-square or Fisher exact test was used to assess the association between ARGs and MGEs in *E. coli* isolates. A *p*-value of <0.05 was considered statistically significant.

## 5. Conclusions

The overall finding of the current study showed the high genetic diversity of ESBL-producing and third-generation cephalosporin-resistant *E. coli* in patients from the two study areas. The observed diversity in the ARGs and MGEs among different STs and serotypes suggests the possible exchange of genes amongst different isolates, posing a significant challenge to infection control and to effectively treating infections. Additionally, a high proportion of self-transferable conjugative elements suggests a significant potential for horizontal gene transfer, with implications for the spread of clinically important traits, such as virulence and antimicrobial resistance. Therefore, the findings from this study highlight the pressing need for comprehensive strategies to address the challenge of AMR in the study area. In the future, based on the findings of this study, enhanced surveillance covering a large geographic area with a representative number of samples in collaboration with various stakeholders and sectors will be proposed to address this growing public health challenge. Implementation of strategies to reduce the spread of ARGs and VAGs is necessary to address this public health threat.

## Figures and Tables

**Table 1:** Distribution of different *E. coli* phylogenetic groups among patients from study sites and their relations with other patients’ characteristics.

Characteristics	Phylogroup (N = 29)						
	A N (%)	B1 N (%)	B2 N (%)	C N (%)	D N (%)	E N (%)	G N (%)
**Study site**							
Addis Ababa (*n* = 11)	7 (63.6)	3 (27.3)	0	0	0	1 (9.1)	0
Hossana (*n* = 18)	2 (11.1)	10 (55.6)	1 (5.6)	2 (11.1)	1 (5.6)	1 (5.6)	1 (5.6)
**Sex**							
Female (*n* = 13)	3 (23.1)	6 (46.2)	1 (7.7)	1 (7.7)	1 (7.7)	1 (7.7)	0
Male (*n* = 16)	6 (37.5)	7 (43.7)	0	1 (6.3)	0	1 (6.3)	1 (6.3)
**Participants**						1 (3.7)	
Diarrheic (*n* = 13)	3 (23.1)	7 (53.8)	0	1 (7.7)	1 (7.7)	1 (7.7)	0
Non-diarrheic (*n* = 16)	6 (35.7)	6 (35.7)	1 (6.3)	1 (6.3)	0	1 (6.3)	1 (6.3)
**Age groups**							
0–4 (*n* = 1)	0	0	0	1 (100)	0	0	0
5–9 (*n* = 6)	2 (33.3)	2 (33.3)	0	0	1 (16.7)	0	1 (16.7)
10–14 (*n* = 3)	1 (66.7)	2 (66.7)	0	0	0	0	0
15–19 (*n* = 2)	1 (50)	1 (50)	0	0	0	0	0
20–45 (*n* = 14)	5 (35.7)	5 (35.7)	1 (7.1)	1 (7.1)	0	2 (14.3)	0
46–65 (*n* = 3)	0	3 (100)	0	0	0	0	0

**Table 2:** Distribution of antimicrobial resistance genes and chromosomal mutations among studied *E. coli* isolates.

Antimicrobial Class	Antimicrobial Resistance Genes/Mutations Detected	No (%) of ESBL-Producing Isolates Possessing the Gene/Mutation (*n* = 19)	No (%) of 3rd-Generation-Cephalosporin-Resistant Isolates Possessing the Gene/Mutation (*n* = 10)	Total No (%) of Isolates Possessing the Gene/Mutation (*n* = 29)
β-lactams	*bla*_CTX-M-15_	15 (78.9)	7 (70)	22 (75.9)
	*bla*_CTX-M-3_	4 (21.1)	0	4 (13.8)
	*bla*_TEM-1B_	10 (52.6)	5 (50)	15 (51.7)
	*bla*_TEM-169_	2 (10.5)	0	2 (6.9)
	*bla*_TEM-33_	2 (10.5)	0	2 (6.9)
	*bla*_SHV-12_	0	1 (10)	1 (3.4)
	*bla*_OXA-1_	1 (5.3)	1 (10)	2 (6.9)
Aminoglycosides	*aph(6)-Id*	8 (42.1)	3 (30)	11 (37.9)
	*aph(3″)-Ib*	8 (42.1)	3 (30)	11 (37.9)
	*aac(6′)-Ib-cr*	1 (5.3)	1 (10)	2 (6.9)
	*aac(3)-IId*	2 (10.5)	0	2 (6.9)
	*aadA1*	2 (10.5)	0	2 (6.9)
	*aadA2*	5 (26.3)	0	5 (17.2)
	*aadA5*	2 (10.5)	2 (20)	4 (13.8)
Quinolones	*qnrS1*	8 (42.1)	5 (50)	13 (44.8)
	*qepA1*	0	1 (10)	1 (3.4)
	*qepA2*	0	1 (10)	1 (3.4)
	*qepA4*	1 (5.3)	1 (10)	2 (6.9)
	*gyrA*:p.S83L	7 (36.8)	4 (40)	11 (37.9)
	*gyrA*:p.D87N	5 (26.3)	3 (30)	8 (27.6)
	*gyrA*:p.S83A	1 (5.3)	0	1 (3.4)
	*gyrA*:p.S83V	1 (5.3)	0	1 (3.4)
	*parC*:p.S80I	5 (26.3)	2 (20)	7 (24.1)
	*parC*:p.E84K	0	1 (10)	1 (3.4)
	*parC*:p.A56T	1 (5.3)	0	1 (3.4)
	*parE*:p.S458A	5 (26.3)	2 (20)	7 (24.1)
	*parE*:p.L416F	0	1 (10)	1 (3.4)
	*parE*:p.I529L	0	1 (10)	1 (3.4)
Sulfonamides	*sul1*	7 (36.8)	3 (30)	10 (34.4)
	*sul2*	8 (42.1)	3 (30)	11 (37.9)
Trimethoprim	*dfrA1*	2 (10.5)	0	2 (6.9)
	*dfrA12*	5 (26.3)	0	5 (17.2)
	*dfrA14*	4 (21.1)	2 (20)	6 (20.7)
	*dfrA17*	2 (10.5)	2 (20)	4 (13.8)
	*dfrB4*	0	1 (10)	1 (3.4)
Tetracyclines	*tet*(A)	9 (47.4)	5 (50)	14 (48.3)
	*tet*(B)	4 (21.1)	1 (10)	5 (17.2)
Macrolides	*mph*(A)	5 (26.3)	2 (20)	7 (24.1)
	*erm*(B)	1 (5.3)	2 (20)	3 (10.3)
Amphenicols	*catA1*	2 (10.5)	1 (10)	3 (10.3)

## Data Availability

The generated sequencing raw data and assembled genomes were submitted to SRA—Sequence Read Archive (accession number: PRJNA1105046, URL https://www.ncbi.nlm.nih.gov/bioproject/PRJNA1105046, accessed on 10 May 2024).

## Supplementary Materials

The following supporting information can be downloaded at: https://www.mdpi.com/article/10.3390/antibiotics13090851/s1, Supplementary Table S1: VAGs detected in *E. coli* isolates; Supplementary Table S2: ARGs detected in *E. coli* isolates; Supplementary Table S3: MGEs detected in *E. coli* isolates.
